# Vitamins as adjunctive treatment for coronavirus disease!

**DOI:** 10.1186/s13613-020-00748-7

**Published:** 2020-09-29

**Authors:** Indu Kapoor, Hemanshu Prabhakar, Charu Mahajan

**Affiliations:** AIIMS, New delhi, India

We read with great interest the article by Li et al., where authors have reviewed many therapeutic strategies for critically ill patients with coronavirus disease (COVID-19) [1]. Apart from antiviral drugs and anti-bacterial drugs, authors also discussed various adjunctive interventions which included corticosteroids, thymosin alpha A, cyclosporine A, interferons, gamma globulins and many more. They even discussed the Chinese medicines which were used in past to treat other viral infections. They also stated that these medicines might benefit COVID-19 patients; however, the efficacy and safety of these traditional medicine formulae in COVID-19 need to be further confirmed by clinical trials. Though authors have not mentioned, another important supplementary adjunct to treat these critically ill patients in intensive care unit is various vitamins.

At present times, good immune system is the major weapon against the COVID-19 and vitamins help in efficient functioning of immune system. Inadequacy of these vitamins in the body can lead to suppressed immunity, which may further predisposes the patient towards infection. These vitamins enhance the three level of immunity in body’s defence system by supporting physical barriers (skin/mucosa), cellular immunity and antibody production. Vitamins like A, C and E help in enhancing the skin barrier function. Vitamins A, B6, B12, C, D and E work synergistically to support the protective activities of the immune cells. All these vitamins except vitamin C are helpful in antibody production. Vitamin A has been shown to enhance immune response against influenza virus [2]. A controlled human trial has reported that Vitamin C significantly lowers the incidence of pneumonia, suggesting that vitamin C may affect susceptibility to lower respiratory tract infections [3]. Kim et al. in their study indicated that combined use of vitamin C, hydrocortisone, and thiamine (vitamin B1) improves the chest radiologic findings of patients with severe pneumonia and reduce their mortality [4]. Ilie et al. identified a negative association between the mean vitamin D levels in various European countries with COVID-19 cases/1M and COVID-19 mortality [5]. The most vulnerable group, the elderly population, is also the amongst the ones who have the most deficient Vitamin D levels and are at higher risk for COVID-19 infection. A large systematic review and meta-analysis including 11,321 patients has also shown that vitamin D supplementation is effective against acute respiratory tract infection [6]. Therefore, these vitamins have shown to protect against the acute viral infections and should be the part of adjunctive therapy in critically ill COVID-19 patients. Despite the use of vitamins worldwide in COVID-19 patients, it is difficult to comment on its absolute therapeutic or preventive role in these group of patients. In future, trials may be needed to understand the role of vitamins in patients of COVID-19.
